# Gain of Chromosome 1q is associated with early progression in multiple myeloma patients treated with lenalidomide, bortezomib, and dexamethasone

**DOI:** 10.1038/s41408-019-0254-0

**Published:** 2019-11-25

**Authors:** Timothy M. Schmidt, Benjamin G. Barwick, Nisha Joseph, Leonard T. Heffner, Craig C. Hofmeister, Leon Bernal, Madhav V. Dhodapkar, Vikas A. Gupta, David L. Jaye, Jiayi Wu, Subir Goyal, Zhengjia Chen, Lawrence H. Boise, Sagar Lonial, Ajay K. Nooka, Jonathan L. Kaufman

**Affiliations:** 1 Winship Cancer Institute, Department of Hematology and Medical Oncology, Emory University, Atlanta, GA USA; 2 Department of Pathology and Laboratory Medicine, Emory University, Atlanta, GA USA; 3 Department of Biostatistics & Bioinformatics, Rollins School of Public Health, Emory University, Atlanta, GA USA

**Keywords:** Myeloma, Cancer genomics, Cancer therapeutic resistance

## Abstract

Gain of chromosome 1q (+1q) is commonly identified in multiple myeloma and has been associated with inferior outcomes. However, the prognostic implication of +1q has not been evaluated in the setting of standard triplet regimens. We retrospectively analyzed 201 consecutive patients with newly diagnosed myeloma who received induction with lenalidomide, bortezomib, and dexamethasone (RVD) and were tested for +1q at diagnosis by fluorescent in-situ hybridization. Patients with +1q (*n* = 94), compared to those without +1q (*n* = 107), had shorter median progression-free survival (PFS) (41.9 months vs 65.1 months, *p* = 0.002, HR = 1.90) and overall survival (median not reached (NR) for either arm, *p* = 0.003, HR 2.69). In subgroup analyses, patients with co-occurring +1q and t(4;14), t(14;16) or del(17p) or with 4 or more copies of 1q had significantly worse PFS (25.1 months and 34.6 months, *p* < 0.001 and *p* = 0.0063, respectively), whereas patients with three copies and no other high-risk cytogenetic abnormalities had no significant difference in PFS. These data suggest that when treated with RVD induction, patients with +1q should be considered at very high risk for early progression in multiple myeloma when ≥4 copies are detected or in the context of other high-risk cytogenetic abnormalities.

## Introduction

Multiple myeloma (MM), a malignant neoplasm of plasma cells, is the second most common hematological malignancy in the Unites States and is characterized by marked clinical heterogeneity and variable outcomes. One of the most important factors determining prognosis for patients with MM is the presence or absence of recurrent chromosomal abnormalities as detected by karyotype and/or fluorescence in-situ hybridization (FISH). It is now well-established that the presence of t(4;14), t(14;16), and/or del(17p) by FISH are associated with early progression and shorter overall survival, and these have been incorporated into the Revised International Scoring System (R-ISS) that predicts survival times for patients with newly diagnosed MM^1^.

Gain of chromosome 1q (+1q) is one of the most common cytogenetic abnormalities in patients with MM, occurring in ~35–40% of patients^2,3^. Many investigators have examined the prognostic implication of +1q, but there remains debate regarding its significance. While some studies have found that +1q is an independent predictor of poor outcomes^2,4–6^, other studies have not shown this correlation^7,8^. Furthermore, these studies have all generally been performed prior to the widespread implementation of novel induction regimens containing the combination of a proteasome inhibitor, immunomodulatory agent, and steroid. Although it has been suggested that bortezomib-based regimens can overcome the negative prognostic impact of +1q^9^, its negative impact on prognosis was maintained in other cohorts^10,11^. Studies that have evaluated the impact of +1q on outcomes of patients treated with novel induction regimens have suggested that +1q remains a poor prognostic factor^12,13^, but conclusions are limited by small sample size and patient selection bias.

It has generally been understood that gain of 1q frequently occurs with disease progression, often by “jumping” translocations and that the copy number can increase over time^14^, with higher rates of +1q detection after progression from the precursor conditions, monoclonal gammopathy of undetermined significance and smoldering multiple myeloma^2^. It has been suggested that any negative impact on survival with +1q may be more profound with amplification of 1q (amp(1q)), defined 4 or more copies of chromosome 1q or with co-occurrence of deletion of chromosome 1p (del(1p))^15,16^. However, the molecular mechanism by which +1q impacts myeloma biology, development, and progression remains poorly understood^17^. Gene expression profiling studies have shown extensive dysregulation of multiple genes on chromosome 1, with a resultant negative impact on prognosis^18^. Overexpression of CKS1B was originally identified as a potential driver gene on 1q^19^, but several other genes of interest including ADAR1 and MCL1 have been suggested more recently^20,21^.

Induction therapy with lenalidomide, bortezomib, and dexamethasone (RVD) is a highly active regimen in MM with substantial progression-free and overall survival benefits for patients over therapy with lenalidomide and dexamethasone alone, and is effective both for patients who proceed to autologous stem cell transplantation (ASCT) or those who are not eligible for ASCT^22,23^. Although this highly active regimen was only recently implemented as standard of care across North America and parts of Europe, RVD has been utilized at our institution for over 12 years. By analyzing our large cohort of patients who were treated uniformly with highly active novel induction therapy, we aimed to determine whether patients with +1q had any distinct clinical features or differences in outcomes compared to patients without +1q.

## Methods

### Patients and demographics

We performed a retrospective analysis of all patients with MM who were seen for an initial visit at Winship Cancer Institute of Emory University between 1 November 2010 and 31 December 2014 and were treated with RVD induction therapy. Patients were identified through an Institutional Review Board-approved myeloma outcomes database that includes all myeloma patients seen at Winship Cancer Institute, with pathological data supplied by the clinical pathology database at Emory University Hospital. Patients in the database were screened by their induction regimen, and patients who were documented as having received induction therapy with RVD were selected for consideration in our analysis. Patients’ electronic health records were reviewed individually in order to confirm their induction treatment and adequacy of diagnostic testing at diagnosis. In order to eliminate the possibility of bias due to clonal cytogenetic evolution seen at relapse, patients were excluded from the analysis if the biopsy report from diagnosis was not available, if it did not have sufficient material for cytogenetic testing, if it was not tested for +1q, or if extra copies of chromosome 1q were only detected by conventional karyotype. Among patients whose diagnostic bone marrows were not enriched for CD138 cells, we included patients if +1q was detected, but excluded those for which +1q was not detected because of uncertainty regarding the presence or absence of 1q gain. A cut-off date for follow up was set at 31 October 2018.

Baseline demographic information (age, sex, race, ethnicity), and laboratory values (hemoglobin, creatinine, calcium, albumin, lactate dehydrogenase (LDH), beta-2-microglobulin (β2M), isotype, M-spike, and serum free light chains) were recorded from the time of diagnosis. Patients were classified by their International Staging System (ISS) stage and Revised International Staging System (R-ISS) at diagnosis. The presence of t(11;14), t(4;14), t(14;16), del(17p), del(13), +1q, and del(1p) were determined by FISH at the time of diagnosis. Patients were also categorized as to whether they had a complex karyotype (defined as three or more abnormalities on conventional cytogenetics). Data regarding date of diagnosis, treatment initiation, ASCT, maintenance therapy, best response to induction therapy and transplantation, and dates of first progression and death were collected. Patient charts were reviewed individually, and data was verified for each patient. External records were reviewed, if available, for patients who were initially diagnosed and/or treated at another medical center.

### Outcomes

The primary outcomes of interest for this study were best response to RVD induction therapy, median PFS, and median overall survival (OS) for patients with +1q compared to patients without +1q. For patients who were treated with autologous stem cell transplantation, responses to transplant and post-transplant PFS and OS were also evaluated. For patients with +1q, additional information was collected and analyzed for copy number (gain(1q), defined as three copies of chromosome 1q, versus amp(1q), defined as four or more copies of 1q) and allele frequency (defined as greater or less than 20% of cells by FISH).

### Statistical analysis

Statistical analysis was conducted using SAS Version 9.4. In the descriptive analysis, *p*-values were calculated using chi-square or Fisher’s exact test. Cox proportional hazard model was employed for the multivariate analysis, including all variables that were significant in the univariate analysis and using backward elimination to exclude variables with *p* ≥ 0.1. To calculate PFS and OS, the Kaplan–Meier method was utilized, and significance between groups was calculated using the log-rank test. A *p*-value of <0.05 was considered as statistically significant for all analyses.

### Analysis of the CoMMpass study

Analysis of the Clinical Outcomes in Multiple Myeloma to Personal Assessment (CoMMpass) trial (NCT01454297) was similar to that previously described^24^. Briefly, outcome data made use of the interim analysis 13 and analysis was restricted to 795 baseline specimens where outcome and long-insert whole genome sequencing data were available in dbGaP (phs000748.v6.p4). Copy number alterations (CNA) at 1q21 were estimated relative to non-myeloma peripheral blood samples using tCoNut (https://github.com/tgen/tCoNuT) as provided in interim analysis 13. Survival analysis made use of the “survival” (v2.43-3) package in R/Bioconductor (v3.6.0). Differences in PFS and OS were determined using a cox proportional hazards regression fit to discrete groups determined by log2 CNA and *p*-values were calculated using a Wald’s test. Relevant clinical and demographic data (age, ISS stage, β2 M levels, M protein levels, gender, race, and front-line therapy) where analyzed between patients with a 1q gain or amplification as compared to those without. Factors that were significantly different in 1q gain or amplified patients were considered in a multivariate analysis with 1q copy number to determine if 1q was an independent prognostic factor.

## Results

**Fig. 1:**
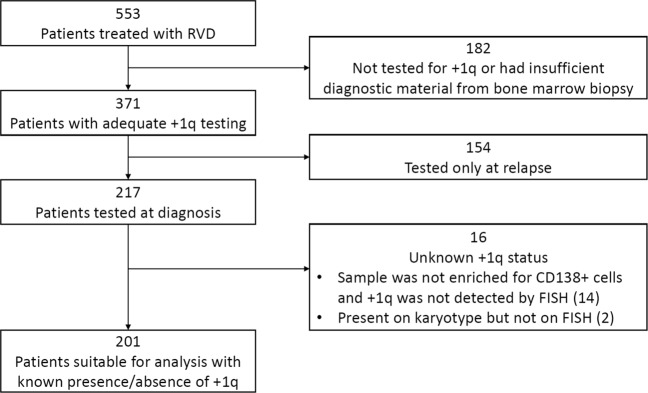
Method of patient selection in final analysis. Flow diagram depicting how patients from the Emory University database were selected for evaluation in the final analysis. Patient records were reviewed individually, induction with RVD was verified, and all pathology reports were reviewed to confirm testing for +1q. Patients were excluded if +1q was tested only at relapse. Patients were also excluded if +1q was not identified on samples not enriched for CD138+ cells or if +1q was detected only by conventional karyotype and not by FISH.

Our search identified 553 consecutive patients who were seen at Winship Cancer Institute of Emory University and were treated with RVD induction for MM. After excluding patients who were not tested for +1q at diagnosis or for whom presence/absence of +1q at diagnosis could not be confirmed, a total of 201 patients were identified as suitable for the final analysis. Methodology by which patients were excluded is detailed in Fig. 1. Of these 201 patients for whom complete diagnostic information was available at diagnosis, 94 (46.7%) had at least one extra copy of chromosome 1q by FISH. Median duration of follow up was 48 months among evaluable patients.

Patient characteristics are summarized in Table 1. Median age at diagnosis was 64 and was not different between groups. Patients with +1q were more likely to present with anemia and/or thrombocytopenia, had higher beta-2-microglobulin, and had a higher ISS and R-ISS stage. Additionally, patients with +1q at diagnosis were more likely to have co-occurrence of t(4;14), t(14;16), del(13q), del(1p), and complex karyotype at diagnosis. There was no significant difference between groups in the frequency of upfront ASCT or whether maintenance therapy was prescribed.

**Table 1:** Patient characteristics.

Characteristic		No +1q		+1q		Total		*P*-value
Age (years)	Median	63		64		64		
	≥ 65	44	41.1%	43	45.7%	87	43.3%	0.509
	< 65	63	58.9%	51	54.3%	114	56.7%	
Sex	Male	64	59.8%	50	53.2%	114	56.7%	0.344
	Female	43	40.2%	44	46.8%	87	43.3%	
Race	African-American	34	31.8%	21	22.3%	55	27.4%	0.186
	Caucasian	63	58.9%	67	71.3%	130	64.7%	
	Unknown/Other	10	9.3%	6	6.4%	16	8.0%	
Isotype	IgG	66	61.7%	45	47.9%	111	55.2%	0.158
	IgA	19	17.8%	27	28.7%	46	22.9%	
	IgD	1	0.9%	0	0.0%	1	0.5%	
	FLC	20	18.7%	20	21.3%	40	19.9%	
	Nonsecretory	1	0.9%	1	1.1%	2	1.0%	
	Oligosecretory	0	0.0%	1	1.1%	1	0.5%	
Hemoglobin (g/dL)	Median	11		10		10.5		
	<10	26	26.8%	39	45.9%	65	35.7%	0.007
	≥10	71	73.2%	46	54.1%	117	64.3%	
Platelets (x10^3^/µL)	Median	218		186.5		207		
	<150	11	12.6%	23	30.3%	34	20.9%	0.006
	≥150	76	87.4%	53	69.7%	129	79.1%	
Creatinine (mg/dL)	Median	1.01		1.03		1.02		
	>2.0	8	8.6%	11	13.6%	19	10.9%	0.294
	≤2.0	85	91.4%	70	86.4%	155	89.1%	
Calcium (mg/dL)	Median	9.2		9.4		9.2		
	>10.5	3	3.6%	13	16.3%	16	9.8%	0.006
	≤10.5	81	96.4%	67	83.8%	148	90.2%	
Albumin (g/dL)	Median	3.7		3.6		3.6		
	<3.5	36	37.9%	35	44.9%	71	41.0%	0.353
	≥3.5	59	62.1%	43	55.1%	102	59.0%	
LDH (units/L)	Median	133		154.5		150		
	>ULN	3	5.0%	5	7.9%	8	6.5%	0.718
	≤ULN	57	95.0%	58	92.1%	115	93.5%	
β2M (mg/L)	Median	2.83		3.8		2.95		
	>5.5	11	11.8%	20	26.7%	31	18.5%	0.014
	≤5.5	82	88.2%	55	73.3%	137	81.5%	
M-spike (g/dL)	Median	1.99		2.7		2.24		
	>3.0	34	37.8%	37	45.1%	71	41.3%	0.329
	≤3.0	56	62.2%	45	54.9%	101	58.7%	
FLC Ratio	>100 or <0.001	42	48.8%	46	60.5%	88	54.3%	0.136
	>0.001 and <100	44	51.2%	30	39.5%	74	45.7%	
ISS	1	43	47.3%	23	30.3%	66	39.5%	0.024
	2	36	39.6%	32	42.1%	68	40.7%	
	3	12	13.2%	21	27.6%	33	19.8%	
R-ISS	1	26	41.9%	14	21.5%	40	31.5%	0.009
	2	34	54.8%	41	63.1%	75	59.1%	
	3	2	3.2%	10	15.4%	12	9.4%	
Upfront Transplant	Yes	88	82.2%	69	73.4%	157	78.1%	0.131
	No	19	17.8%	25	26.6%	44	21.9%	
Maintenance	Yes	83	79.0%	72	80.0%	155	79.5%	0.870
	No	22	21.0%	18	20.0%	40	20.5%	
t(11;14)	No	81	77.9%	76	86.4%	157	81.8%	0.129
	Yes	23	22.1%	12	13.6%	35	18.2%	
t(4;14)	No	103	99.0%	81	91.0%	184	95.3%	0.013
	Yes	1	1.0%	8	9.0%	9	4.7%	
t(14;16)	No	103	99.0%	79	90.8%	182	95.3%	0.012
	Yes	1	1.0%	8	9.2%	9	4.7%	
del(17p)	No	97	90.7%	76	84.4%	173	87.8%	0.184
	Yes	10	9.3%	14	15.6%	24	12.2%	
del(13q)	No	79	74.5%	33	36.7%	112	57.1%	<0.001
	Yes	27	25.5%	57	63.3%	84	42.9%	
Hyperdiploidy	No	35	33.7%	34	39.5%	69	36.3%	0.401
	Yes	69	66.3%	52	60.5%	121	63.7%	
del(1p)	No	99	94.3%	73	83.0%	172	89.1%	0.012
	Yes	6	5.7%	15	17.0%	21	10.9%	
Complex Karyotype	No	92	86.0%	63	68.5%	155	77.9%	0.003
	Yes	15	14.0%	29	31.5%	44	22.1%	

### Response rates and survival outcomes

**Fig. 2:**
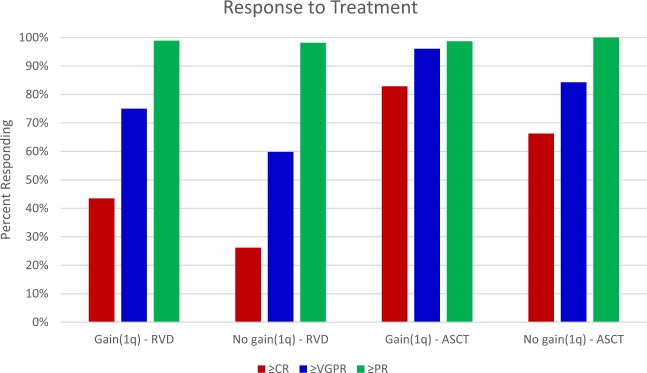
Best response to RVD and ASCT. Percentage of patients achieving at least a complete response (≥CR), very good partial response (≥VGPR), and partial response (≥PR) to RVD induction and ASCT is demonstrated. Patients were grouped by presence or absence of +1q and best response after completion of induction or transplant.

Best response to RVD induction is shown in Fig. 2. Overall response rate was similar for patients with +1q and patients without +1q, (98.9% vs 98.1%). However, patients with +1q had significantly deeper responses; in particular, patients with +1q were more likely to achieve a VGPR or better compared to patients without +1q (75.0% vs 59.8%, *p* = 0.02).

**Fig. 3:**
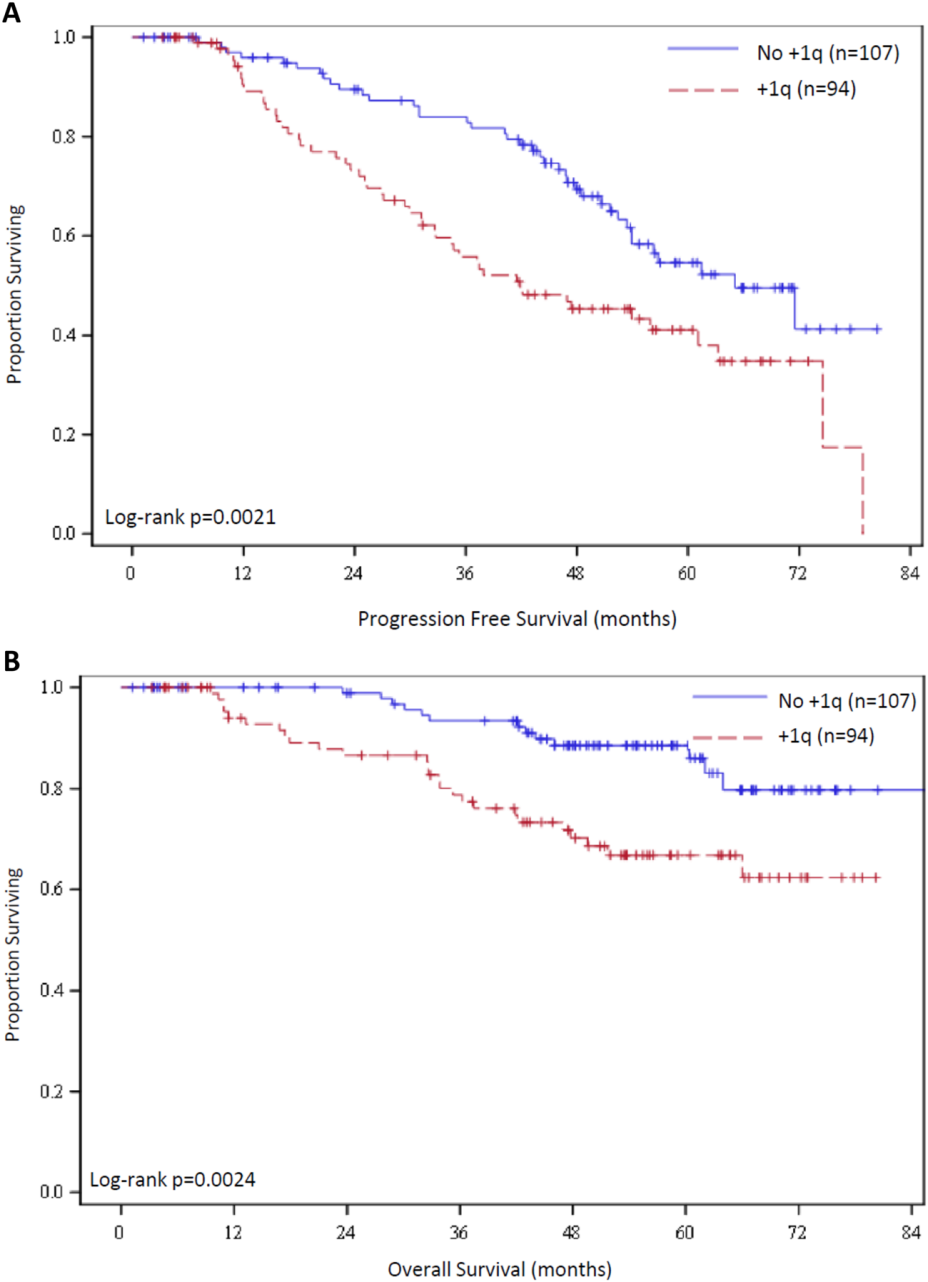
Progression-free and overall survival. Kaplan–Meier curves are shown for **a** PFS and **b** OS for patients with +1q and without +1q.

Kaplan–Meier curves for PFS and OS are shown in Fig. 3. Median PFS for patients with +1q patients was 41.9 months (95% CI 32.6–63.3 mo) compared with 65.1 months (95% CI 53.4 mo–NR) among patients who did not have +1q (*p* = 0.002, HR 1.91). Patients with +1q had a 5-year PFS rate of 41.3% (95% CI 29.9–52.4%) compared to 54.6% (42.4–65.3%) for patients without +1q. Median OS was NR in either group, but Kaplan–Meier curves had clear early separation and patients with +1q had significantly inferior OS rates compared to those without +1q (*p* = 0.0024, HR 2.69). Five-year OS rate for patients with +1q was 66.9% (95% CI 54.9–76.4%), which is significantly lower than the 5-year OS rate of 88.5% (95% CI 79.7–93.7%) among patients without +1q.

On univariate analysis, calcium > 10.5 mg/dL, lack of maintenance therapy, t(4;14), t(14;16), del(17p), del(13q), complex karyotype, and +1q were identified as factors significantly associated with worse PFS. Comprehensive results from univariate analysis are shown in Table 2. On multivariate analysis, +1q retained its negative impact on PFS (*p* = 0.018, HR 1.89, 95% CI 1.11–3.20). Other factors that were significant in multivariate analysis included maintenance therapy, which was associated with significantly improved PFS, as well as calcium >10.5, t(4;14), t(14;16), and del(17p) which were all significantly associated with worse PFS (Table 3).

**Table 2:** Univariate analysis.

Characteristic		*N*	Hazard ratio (95% CI)	HR *P*-value	Log-rank *P*-value
Age	≥65	114	0.79 (0.52–1.20)	0.264	0.262
	<65	87	Ref		
Sex	Male	114	1.15 (0.75–1.76)	0.514	0.513
	Female	87	Ref		
Race	African-American	55	0.89 (0.54–1.46)	0.644	0.743
	Caucasian	130	Ref		
	Other/Unknown	16	0.73 (0.29–1.82)	0.504	
Isotype	IgG	111	Ref		0.171
	IgA	46	1.12 (0.68–1.87)	0.654	
	FLC	40	0.96 (0.55–1.68)	0.891	
	Others	4	3.44 (1.06–11.14)	0.04	
Hemoglobin	<10	65	1.27 (0.80–2.00)	0.311	0.309
	≥10	117	Ref		
Platelets	<150	34	1.32 (0.74–2.36)	0.34	0.338
	≥150	129	Ref		
Creatinine	>2.0	19	1.70 (0.87–3.31)	0.122	0.117
	≤2.0	155	Ref		
Calcium	>10.5	16	3.29 (1.68–6.47)	<0.001	<0.001
	≤10.5	148	Ref		
Albumin	<3.5	71	0.75 (0.46–1.22)	0.245	0.242
	≥3.5	102	Ref		
LDH	<ULN	115	1.06 (0.26–4.38)	0.934	0.934
	≥ULN	8	Ref		
β2M	>5.5	31	1.15 (0.66–2.00)	0.63	0.629
	≤5.5	137	Ref		
M-spike	>3.0	71	0.92 (0.58–1.47)	0.741	0.741
	≤3.0	101	Ref		
FLC Ratio	>100 or <0.001	88	1.48 (0.90–2.42)	0.122	0.119
	0.001 < k/l < 100	74	Ref		
ISS	1	66	Ref		0.966
	2	68	1.00 (0.60–1.67)	0.995	
	3	33	1.08 (0.59–1.98)	0.813	
R-ISS	1	40	Ref		0.294
	2	75	1.27 (0.70–2.30)	0.44	
	3	12	2.10 (0.82–5.39)	0.124	
Upfront transplant	Yes	157	0.71 (0.41–1.24)	0.226	0.223
	No	44	Ref		
Maintenance	Yes	155	0.49 (0.29–0.83)	0.008	0.007
	No	40	Ref		
t(11;14)	Yes	35	0.86 (0.49–1.54)	0.619	0.618
	No	157	Ref		
t(4;14)	Yes	9	2.45 (1.06–5.63)	0.035	0.029
	No	184	Ref		
t(14;16)	Yes	9	2.77 (1.12–6.88)	0.028	0.022
	No	182	Ref		
del(17p)	Yes	24	2.53 (1.40–4.59)	0.002	0.001
	No	173	Ref		
del(13q)	Yes	84	1.71 (1.13–2.60)	0.011	0.01
	No	112	Ref		
Hyperdiploidy	Yes	121	0.73 (0.47–1.15)	0.174	0.172
	No	69	Ref		
del(1p)	Yes	21	1.27 (0.66–2.46)	0.473	0.472
	No	172	Ref		
Complex Karyotype	Yes	44	2.05 (1.30–3.22)	0.002	0.002
	No	155	Ref		
Gain(1q)	Yes	94	1.91 (1.26–2.91)	0.003	0.002
	No	107	Ref

**Table 3:** Multivariate analysis.

		*N*	Hazard ratio (95% CI)	HR *P*-value
Calcium	>10.5	16	2.34 (1.04–5.28)	0.04
	≤10.5	148	Ref	
Maintenance	Yes	155	0.45 (0.22–0.92)	0.029
	No	40	Ref	
t(4;14)	Yes	9	4.18 (1.46–11.96)	0.008
	No	184	Ref	
t(14;16)	Yes	9	2.80 (1.07–7.34)	0.036
	No	182	Ref	
del(17p)	Yes	24	2.52 (1.25–5.09)	0.01
	No	173	Ref	
Gain(1q)	Yes	94	1.92 (1.14–3.25)	0.015
	No	107	Ref	

Number of observations in the original data set = 201. Number of observations used = 149. Backward selection with an alpha level of removal of 0.10 was used. The following variables were removed from the model: Complex Karyotype and del(13q)

### Outcomes of patients undergoing upfront ASCT

Seventy-six of 94 (80.9%) patients with +1q and 89 of 107 (83.1%) patients without +1q underwent ASCT. Among the 76 patients with +1q who did receive a transplant, 73 (96.1%) of them achieved a VGPR or better compared to 75 of 89 (84.3%) patients achieving a VGPR after transplant among patients who did not have +1q (*p* = 0.01). As seen in Fig. 2, the difference between groups was primarily driven by the high rate of CR (82.9%) among patients with +1q compared to those without +1q (66.3%). Despite the improved response rates, median PFS was significantly shorter among transplanted patients with +1q (42.1 months; 95% CI 32.8–78.9 mo) compared to transplanted patients without +1q (71.5 months; 95% CI 52.5mo–NR) (*p* = 0.0136). Median OS was NR for either arm, but five-year OS after transplant was 66.8% (95% CI 53.4–77.1%) for patients with +1q compared to 88.4% (95% CI 78.9–93.8%) for patients without +1q (*p* = 0.006).

### Impact of co-occurring cytogenetic abnormalities

**Fig. 4:**
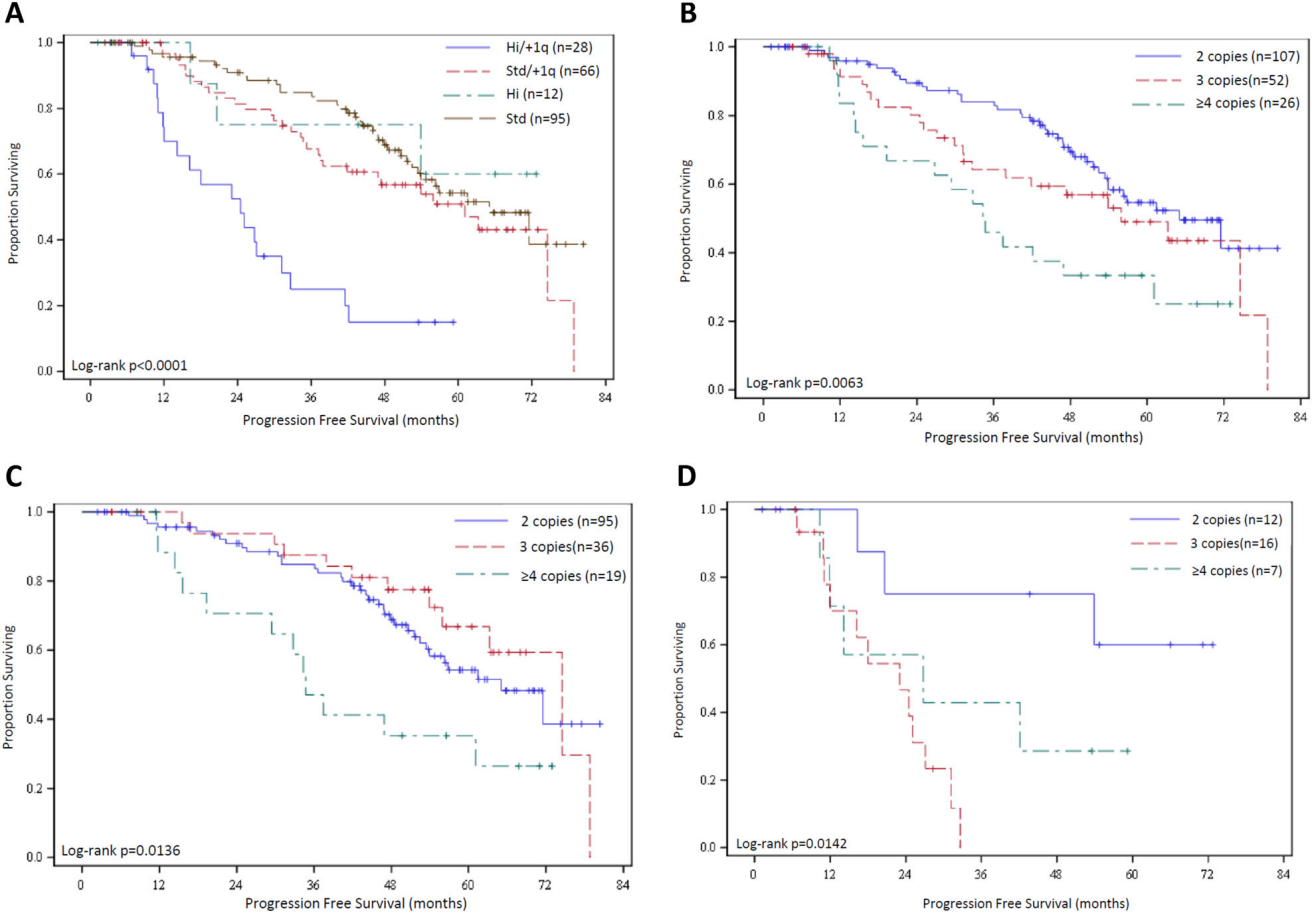
Pertinent subgroup analyses. **a** Kaplan–Meier curves are shown for patients as stratified by their cytogenetics (high risk, “Hi,” defined as t(4;14), t(14;16) and/or del(17p) or standard risk, “Std”) and presence or absence of +1q. HR for Hi/+1q vs Std/+1q = 3.34 (1.83-6.08); Hi/+1q vs Hi = 3.97 (1.14-13.81); Std/+1q vs Std = 1.39 (0.86-2.24). **b** Kaplan–Meier curves for patients stratified by copy number of chromosome 1q by FISH. HR for 3 vs 2 copies = 1.47 (0.88-2.45); 4 vs 2 copies = 2.45 (1.38-4.35); 4 vs 3 copies = 1.67 (0.89-3.12). **c** PFS of standard risk patients stratified by 1q copy number. HR for 2 vs 4 copies = 0.44 (0.23-0.84); 3 vs 4 copies = 0.34 (0.15-0.76); 2 vs 3 copies = 1.28 (0.66-2.46). **d** PFS of high-risk patients stratified by 1q copy number. HR for 2 vs 4 copies = 0.31 (0.07-1.32), 3 vs 4 copies = 2.06 (0.67-6.29); 2 vs 3 copies = 0.15 (0.04-0.61).

Among the 201 patients in this series, 40 (19.9%) were identified as high-risk by consensus criteria (defined by the presence of t(4;14), t(14;16), and/or del(17p) by FISH). Among high-risk patients, +1q was also identified in 28 (70% of high-risk patients). Median PFS of high-risk patients also harboring +1q was 25.1 months (95% CI 12.0–32.6 mo), which was significantly worse than high-risk patients who did not have +1q (*p* = 0.02). Patients with +1q who did not have other high-risk cytogenetic abnormalities (*n* = 66) had a median PFS of 61.1 months (95% CI 37.9–78.9 mo), which is significantly better than those with high risk and +1q (*p* < 0.001). The median PFS of the 95 patients without high-risk cytogenetics or +1q was 65.1 months (95% CI 52.5 mo–NR), which is not significantly different compared to the PFS of patients with +1q but no other high-risk cytogenetic abnormalities (*p* = 0.1817). These findings are demonstrated in Fig. 4a.

### Impact of copy number and detection threshold

Among patients with +1q, we sought to determine whether 1q copy number, percentage of cells with +1q by FISH, and/or co-occurrence of del(1p) impacted outcomes among patients with +1q. Among the 94 patients with +1q, 78 had copy number quantified in the FISH report. 52 (66.7%) had only one additional copy identified at the time of diagnosis (three copies) and 26 (33.3%) had two or more extra copies of chromosome 1q (four or more copies/amp(1q)). There were no significant differences in baseline characteristics or cytogenetics between patients with amp(1q) compared to those with gain(1q) (Supplementary Table 1). Patients with three copies of 1q had a median PFS of 55.9 months (32.6–78.9 mo) and those with amp(1q) had a median PFS of 34.6 months (15.6–61.1 months). When compared to the median PFS of 65.1 months for the cohort without +1q, these outcomes were significantly different (*p* = 0.0063), as demonstrated in Fig. 4b. Impact of 1q copy number by cytogenetic risk is shown in Fig. 4c, d. Among patients with standard risk cytogenetics, those with 2, 3, or ≥4 copies had a median PFS of 65.0 months (95% CI 52.4 mo–NR), 74.5 months (55.9–78.8 mo), and 34.7 months (15.6–61.1 mo), respectively. Among patients with high-risk cytogenetics, median PFS for patients with 2, 3, or ≥4 copies was NR (16.3mo–NR), 23 months (11.0–27.1 mo), and 26.8 months (10.3 mo–NR).

There was no significant difference in PFS among patients with 20% or more cells positive for +1q compared to those with <20% by FISH (*p* = 0.1837, data not shown). Of 88 patients with +1q who were also tested for del(1p), 15 patients also had del(1p) by FISH at the time of diagnosis (17.0%), but there was no difference in PFS between these patients and those with +1q alone (*p* = 0.9753, data not shown).

### Impact of Chromosome 1q copy number alteration in the CoMMpass Trial

We sought to validate our findings using an independent dataset and thus made use of the Multiple Myeloma Research Foundation’s CoMMpass trial, which is a large, multinational, prospective cohort study that enrolled 1150 patients and analyzed the majority of samples using multiple genomic technologies. We restricted our analysis to 870 newly diagnosed patients with outcome data and 1q21 copy number alterations (CNA) derived from whole genome long-insert sequencing. Comparison of demographics, baseline clinical characteristics, induction regimens, of CoMMpass patients with +1q compared to those without +1q is demonstrated in Supplementary Fig. 1A–G. Similar to patients treated at Emory, CoMMpass patients with +1q tended to have a higher ISS stage, age, and beta-2-microglobulin levels.

**Fig. 5:**
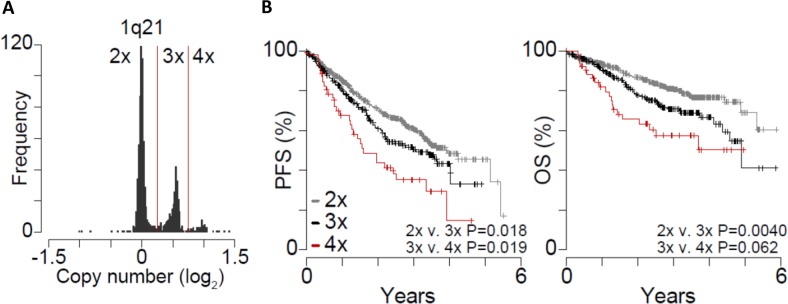
In CoMMpass, +1q21 corresponds with poor prognosis. **a** Copy number alterations at the 1q21 locus in 870 myeloma samples from newly diagnosed patients in the CoMMpass study (IA13). Copy number is plotted as the log2 ratio of myeloma compared to normal from the same patient. Copy number thresholds are denoted by red lines and labeled (top). **b** Progression-free survival (PFS; left) and overall survival (OS; right) for patients with myeloma that has 2 × (gray; *N* = 575), 3 × (black; *N* = 242), and 4 × (red; *N* = 53) 1q21 copies. *P*-values indicate the difference in survival between 2×, 3×, and 4× 1q21 copies as determined using a Cox Proportional Hazards Wald test.

Analysis of 1q21 copy number represented as a log_2_ ratio relative to normal peripheral blood from the same patient, clearly delineated patients with two copies (log ratio ~ 0) of 1q21 from those with three (log ratio ~ 0.5) or four copies (log ratio ~ 1) (Fig. 5a). Of the 870 evaluable patients in the CoMMpass database, 575 (66.1%) did not have +1q and 295 (33.9%) had +1q. Among patients with +1q, 242 (82.0%) had one additional copy and 53 (18.0%) had two or more additional copies of 1q. Survival analysis of these patients indicated that three copies of 1q had significantly inferior PFS and OS compared to those with two copies (*p* = 0.018 and *p* = 0.004, respectively). Patients with four or more copies of 1q had significantly worse PFS compared to those with three copies (*p* = 0.019) and had a trend towards inferior OS (*p* = 0.062) (Fig. 5b). Importantly, +1q remained significant on multivariate analysis when controlling for differences in clinical data among CoMMpass patients (Supplementary Fig. 1H). These data support our observations that reduced PFS and OS occur with increasing 1q amplification.

## Discussion

In this study, we sought to characterize the clinical characteristics and outcomes of patients with multiple myeloma harboring +1q at diagnosis in the era of novel induction therapy. Although large, prospective studies have been performed in the past with attention to the impact of +1q on outcomes^2,6,25^, the relevance of the prognostic impact of +1q in those studies uncertain as these older regimens including thalidomide and/or chemotherapy are not widely used in current myeloma treatment regimens. RVD has become the new standard of care in the United States based on its remarkable efficacy in both transplant-eligible and ineligible patients^22,23^. Our analysis is the largest to date that evaluates the prognostic impact of +1q in patients treated with RVD.

In our analysis, we found that among patients who were tested for the presence of +1q by FISH at diagnosis, +1q was seen 46.7% of newly diagnosed cases. Compared to patients without +1q, those with +1q at diagnosis were more likely to present with anemia, thrombocytopenia, high disease burden as signified by beta-2-microglobulin, and higher ISS stage. Additionally, +1q was highly associated with the co-occurrence of high-risk cytogenetic abnormalities including t(4;14), t(14;16) as tested by FISH, and complex karyotype by conventional karyotype, however there was no significant difference in co-occurrence of del(17p).

We found in our analysis that patients with +1q had a significantly higher chance of achieving a VGPR or CR with induction therapy. Despite these deeper responses, the presence of +1q was associated with significantly shorter PFS and OS compared to patients without +1q. This impact on prognosis remained significant even among patients who underwent consolidation with ASCT in first remission, despite 96% of patients with +1q achieving a VGPR or better after ASCT. We noted a significant decrease in PFS with increasing copy number of +1q, as shown in Fig. 4b, with remarkably similar findings among patients in CoMMpass (Fig. 5b). Whether +1q is itself a driver of these inferior outcomes is unclear, as there was no significant difference in PFS between standard risk patients with or without gain of one copy of 1q (Fig. 4c). However, all patients with amp(1q) and high-risk patients with +1q, regardless of copy number, had substantial reduction in PFS (Fig. 4c, d). As seen in Fig. 4a, the data suggest that standard risk patients with +1q and high-risk patients without +1q may have an intermediate risk of progression when treated with RVD induction, but our dataset was underpowered to make this conclusion. To test this hypothesis, we performed the same analysis within the CoMMpass database and found similar findings, as shown in Supplementary Fig. 2. These results suggest that the combination of +1q and another high-risk CA or amp(1q) effectively result in a “ultra-high risk” subgroup that has dismal outcomes despite excellent response rates to RVD and high rates of ASCT.

Many other attempts have been made to risk stratify patients who are at high risk of early disease progression in order to identify patients who should be considered for novel treatment approaches. Recently, the concept of “double hit” myeloma has been proposed^26^. In a large, international, cooperative study, whole-exome sequencing (WES) was performed in an attempt to identify genetic predictors of early disease relapse. In this study, the authors identified two populations at very high risk of early progression or death—the presence of bi-allelic inactivation of TP53 (by loss and/or mutation) and patients with 4 or more copies of CKS1B with ISS stage 3 disease. Notably, this study did not stratify patients by induction regimen. When evaluating only patients who were treated with RVD induction, our study complements the existing data that amp(1q) identifies a very high-risk patient population, and suggests that co-occurrence of +1q and t(4;14), t(14;16), or del(17p) may be another category of “double hit” patients who are at high risk for early progression despite aggressive treatment.

Our approach to the management of high-risk patients generally includes a risk-adapted maintenance strategy. We previously reported PFS and OS rates markedly superior to traditional benchmarks in high-risk patients with use of RVD maintenance for patients with t(4;14), t(14;16), del(17p) and/or complex karyotype^27^. This approach highlights the fact that high-risk patients can achieve durable remissions if an aggressive approach is employed and that the depth of response is maintained for as long as possible. In this subset of patients, progressive disease leads to a vicious cycle of early progression on second-line therapies and beyond, with very few patients achieving durable second remissions. The current analysis demonstrates that +1q, when present with a high-risk cytogenetic abnormalty, is associated with dismal outcomes, with a median PFS of only 25.1 months, compared to median PFS in excess of 60 months for all subgroups of patients without both factors. Of the 28 patients with +1q and additional high-risk CA, 20 (71%) were treated with maintenance therapy and 14 of these patients received some sort of risk-adapted maintenance therapy. While this subset is too small to draw any significant conclusions regarding the benefit of risk-adapted maintenance, particularly within the retrospective nature of this analysis, the data suggest that patients with +1q and co-occurrence of other high-risk cytogenetic abnormalities may progress early despite risk-adapted maintenance, and this may be a unique subset of patients with multiple myeloma that should be considered for novel treatment approaches early in their disease course.

In the IFM2009 trial^22^, a pre-planned analysis of outcomes according to minimal residual disease (MRD) was performed for patients who were treated with RVD induction and then randomized to ASCT or no transplant. In this analysis, it was clearly demonstrated that MRD negativity (at a depth of 10^−5^) by next generation sequencing was a more powerful prognostic marker than cytogenetic risk, as patients with high-risk cytogenetics with MRD negativity had markedly better PFS than standard-risk patients who were MRD positive^28^. Although information about MRD status was not available for the patients in our data set, the very high CR rate after transplant among patients with +1q raises the question of whether MRD is as powerful of a prognostic factor in these patients. Alternatively, it is possible that although RVD and ASCT are very effective at inducing a CR for +1q patients, MRD negativity could be more difficult to achieve in these patients. Notably, +1q data is not available from the IFM manuscripts, and this remains an unanswered question that should be evaluated in future analyses.

The primary strengths of this study include our large number of consecutive patients treated uniformly with RVD induction and a long duration of follow up, with restriction of patients to only those with high-quality diagnostic information. Importantly, our dataset is made up of a diverse patient population, which includes 31.8% African-American patients. This contrasts to other large datasets that have generally under-represented patients of African descent and makes our analysis more widely applicable. Our analysis is limited by the single-center, retrospective nature of the data collection, and the outcomes seen in this patient population may be biased by the fact that most patients are healthy enough to be considered for transplantation as the reason for referral to our center. Because we are largely a referral center, much of the data regarding labs and pathology at diagnosis is dependent on documentation by referring providers, resulting in missing data from the time of diagnosis. In particular, data for LDH and beta-2-microglobulin among patients referred from other medical centers were often missing and this may explain the lack of prognostic value of the ISS and R-ISS in our univariate analysis. However, the fact that we were able to show similar results for PFS among patients with +1q within CoMMpass, a large, prospective, multinational study, helps to validate our findings and broaden the applicability of our study. Of note, CoMMpass results must be interpreted with caution due to the heterogeneity of induction regimens used among these patients. Importantly, our analysis did not find confounding treatment regimens between +1q and other patients in CoMMpass (Supplementary Fig. 1).

In conclusion, patients with multiple myeloma who have +1q by FISH are more likely than those without +1q to present with anemia, thrombocytopenia, hypercalcemia, higher ISS stage, and co-occurrence of high-risk cytogenetic abnormalities. Patients with +1q myeloma have significantly inferior PFS and OS compared to patients who do not have +1q despite having deeper responses to RVD induction therapy and ASCT. Although PFS is negatively impacted by the addition of one copy of 1q, having two or more additional copies of 1q at diagnosis is associated with significantly inferior PFS and OS compared to patients with only 3 copies of 1q. There is no significant impact on PFS or OS among standard-risk patients with gain(1q), but patients with +1q and any high-risk cytogenetic abnormality and patients with amp(1q), regardless of other cytogenetics, have extremely short PFS. Patients with amp(1q) and/or co-occurrence of +1q and high-risk cytogenetic abnormalities should be considered at very high risk for early progression or death and should be considered for more aggressive management and/or clinical trials early in their disease course.

## Supplementary information

Supplementary Figure 1

Supplementary Figure 2

Supplementary Table 1
