# Supplementary material for: Gain of Chromosome 1q is associated with early progression in multiple myeloma patients treated with lenalidomide, bortezomib, and dexamethasone
Source: Blood Cancer J. 2019 Nov 25;9(12):94. doi: 10.1038/s41408-019-0254-0 (PMC6877577; doi:10.1038/s41408-019-0254-0)
Supplement: Supplementary file 1 — Supplementary Figure 1 [file 41408_2019_254_MOESM1_ESM.docx]

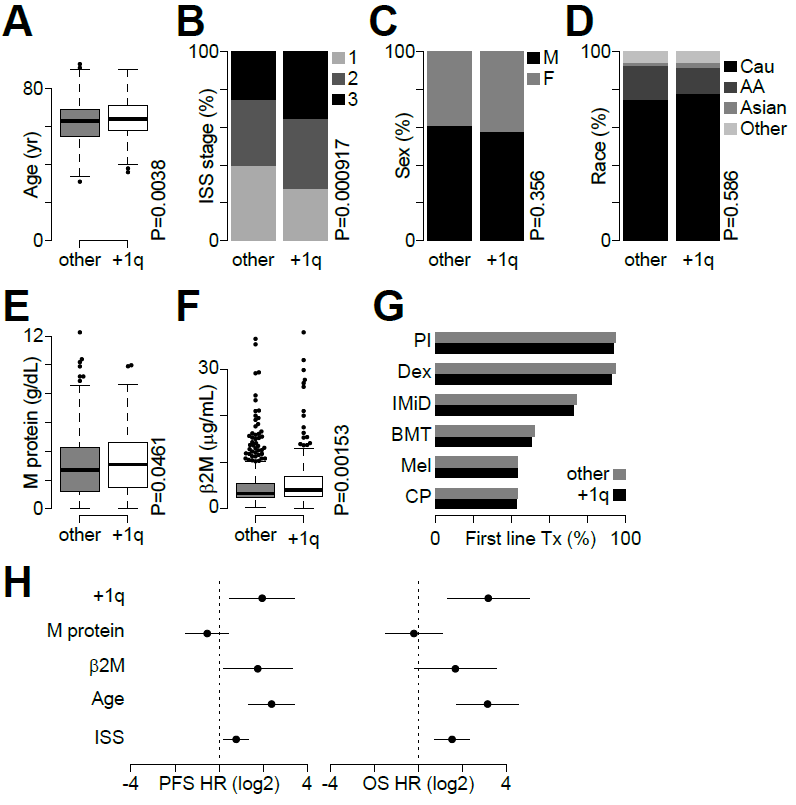


**Supplementary Figure 1.** Copy number alterations in chromosome 1q (+1q) are indicative of poor prognosis. **A)** Age, **B)** ISS stage, **C)** sex, **D)** race, **E)** M protein, and **F)** β-2-microglobulin (β2M) distribution of patients with (+1q) and without (other) a 1q gain or amplification. **G)** Front-line therapies used to treat patients with and without a 1q gain or amplification. **H)** Hazard ratios (HR) determined by multivariate analysis of 1q gain or amplification, M protein, β2M, age, and ISS stage. Boxplots (**A**, **E**, **F**) show the median and quartiles with the whiskers extending to the most extreme data point within 1.5 times the interquartile ran. P-values were calculated by Mann-Whitney U-test (**A**, **E**, **F**) or Fisher’s exact test (**B**, **C**, **D**). Hazard ratios where based on scaled values (0-1) for each covariate (**H**). Data are from CoMMpass IA13.
