# Supplementary material for: Gain of Chromosome 1q is associated with early progression in multiple myeloma patients treated with lenalidomide, bortezomib, and dexamethasone
Source: Blood Cancer J. 2019 Nov 25;9(12):94. doi: 10.1038/s41408-019-0254-0 (PMC6877577; doi:10.1038/s41408-019-0254-0)
Supplement: Supplementary file 2 — Supplementary Figure 2 [file 41408_2019_254_MOESM2_ESM.docx]

** Supplementary Figure 2**. progression-free (PFS; left) and overall survival (right) of patients with standard-risk (**Std**) or high-risk genetics [**Hi**; t(4;14), t(14;16), or del(17p)] as well as with a chromosome 1q (**1q**) gain or amplification. P-values and hazard ratios (**HR**) for differences between groups are denoted on the right as calculated using a Cox proportional hazards regression Wald test with 95% confidence intervals denoted in parenthesis.
